# Hydrogels on the Base of Modified Chitosan and Hyaluronic Acid Mix as Polymer Matrices for Cytostatics Delivery

**DOI:** 10.3390/gels8020104

**Published:** 2022-02-09

**Authors:** Regina Vildanova, Alexander Lobov, Leonid Spirikhin, Sergey Kolesov

**Affiliations:** Ufa Institute of Chemistry, Ufa Federal Research Centre of the Russian Academy of Sciences, 450054 Ufa, Russia; lobovan@anrb.ru (A.L.); spectr@anrb.ru (L.S.); kolesovservic@rambler.ru (S.K.)

**Keywords:** chitosan, cross-linking, cytostatic, drug delivery, hyaluronic acid, hydrogel, Schiff base

## Abstract

The development of biodegradable polysaccharide hydrogel matrices for cytostatic delivery can improve the therapeutic results of patients by prolonging the action of the drug, reducing its toxicity and providing additional biological activity by polysaccharides. In this work, N-succinyl chitosan/hyaluronic acid dialdehyde/cytostatic formulations have been prepared using two different chitosan grades (30 kDa and 150 kDa) and hyaluronic acid dialdehyde. The interaction of amino groups of N-succinyl chitosan and aldehydes of hyaluronic acid resulted in the formation of azomethine bonds and was demonstrated using ^13^C NMR. The elastic properties of the obtained hydrogels determine their use as implants. Two cytostatics—5-fluorouracil and mitomycin C were chosen as drugs because of their using both in oncology and in ophthalmology for the surgical treatment of glaucoma. Hydrogel formulations containing cytostatic were prepared and drug release was studied using in vitro dialysis method. It was established that the molecular weight of N-succinyl chitosan and rheological properties of hydrogel influenced the drug release behavior of the gelling delivery system. Formulations prepared from N-succinyl chitosan with greatest molecular weight and mitomycin C were found to be the most promising for medical application due to their rheological properties and prolonged drug release. Mild preparation conditions, simplicity of the technique, short gelation time (within a minute), 100% yield of hydrogel, suitability for drug release applications are the main advantages of the obtained hydrogels.

## 1. Introduction

Hydrogel represent three-dimensional network structure, consisting of chemically or physically cross-linked polymers, holding large amounts of liquid in its meshes. Their unique properties, including high water absorption, tunable mechanical features and sensitivity to various stimuli has made them important candidates in biomedical applications including drug delivery. The use of hydrogels or aerogels as drug carriers provides its prolonged action under certain conditions, which reduces the side effects of the drug and improves the effectiveness of treatment, thereby improving the quality of patient life [1,2,3,4,5,6].

Most of the hydrogels on the market are based on synthetic polymers. The use of natural polymers, such as chitosan and hyaluronic acid, allows the transfer of bioactive properties of polysaccharides to the polymer matrix: biodegradability, biocompatibility, mucoadhesiveness, antibacterial and anti-inflammatory properties. In terms of their microstructure, hydrogels are similar to the intercellular matrix of many tissues and are capable of imitating its physicochemical properties. Thus, they are an ideal cellular microenvironment for cell proliferation and differentiation [7,8,9,10,11,12].

The simple and effective method of chemical cross-linking without use of cross-linkers is associated with the Schiff base reaction, which occurs due to the formation of imine bonds in mild conditions (pH = 7.4), which are biodegradable due to hydrolysis [13,14]. The use of two types of polymers containing amino groups and aldehyde groups makes it possible to carry out a cross-linking reaction without the use of additional organic reagents.

Chitosan is a copolymer of 2-acetamido-2-deoxy-β-D glucopyranose and 2-amino-2-deoxy-β-D glucopyranose, obtained by the thermochemical deacetylation of chitin under basic or enzymatic conditions. Chitin is among the most abundant natural polysaccharides, found in exoskeletons of crustaceans, insects, and certain fungi but its applications are limited due to the poor solubility in organic and aqueous media. However, using thermochemical deacetylation under basic or enzymatic conditions, chitin can be converted into chitosan that is soluble in acidic media (below pH 6.0) but limits its use for the preparation of hydrogels at neutral pH values. N-succinyl chitosan (SCTS) is an amphiprotic derivative obtained from the N-acylation of chitosan. SCTS exhibits extraordinary biocompatibility, significantly increased aqueous solubility in acidic and basic media without affecting the biological properties, appreciable transfection efficiency, and the ability to stimulate osteogenesis [11,15].

Hyaluronic acid (HA) is a non-sulphated glycosaminoglycan, composed of alternating units of D-glucuronic acid and N-acetyl-D-glucosamine. HA is presented in the extracellular matrix of many soft connective tissues, including eye vitreous humor. Native high molecular weight hyaluronic acid forms viscous hydrogels due its high hydrophilicity, but it undergoes rapid degradation under the action of enzymes in the body. Therefore, it needs to be modified using chemical cross-linking to obtain stable hydrogels [16,17].

Previously, we have prepared and investigated the rheological properties of hydrogels on the base of N-succinyl chitosan and hyaluronic acid dialdehyde [18]. In addition, this current work is devoted to possibility of preparation hydrogels based of N-succinyl chitosan and hyaluronic acid dialdehyde (HAD), containing cytostatics in their dispersion medium, the release rate of which is regulated by the molecular weight of chitosan.

## 2. Results and Discussion

### 2.1. SCTS Synthesis and Characterization

**Scheme 1:**
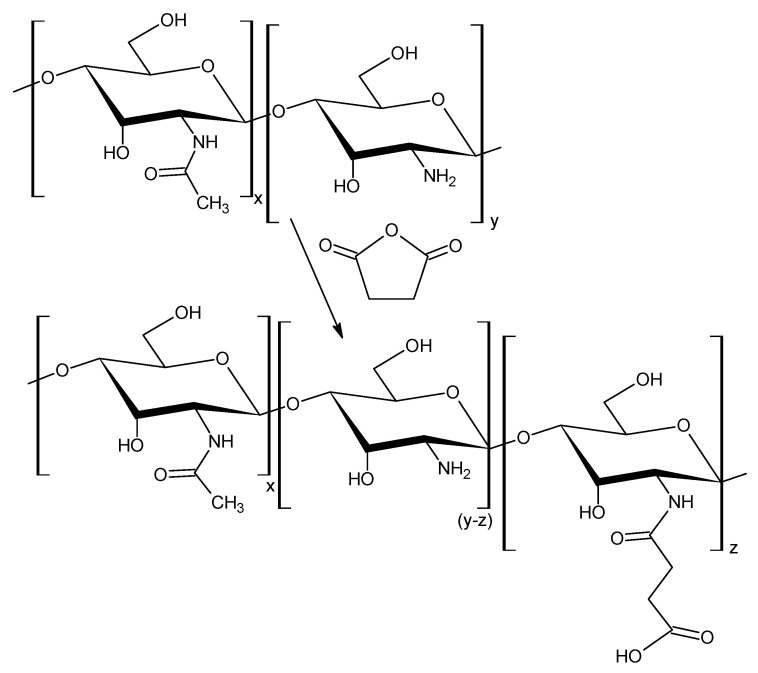
Synthesis of N-succinyl chitosan.

**Figure 1:**
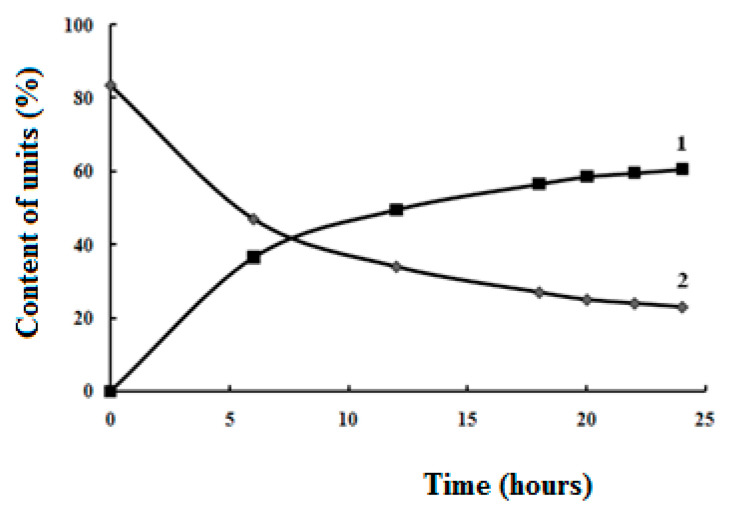
Kinetic curves of the accumulation of units containing carboxyl groups (1) and the consumption of units containing amino groups (2) in relation to the total content of units containing amino groups in the native chitosan.

It is known [11], that native chitosan does not dissolve in water, which is some obstacle for the preparation of hydrogels in a neutral medium (pH ≈ 7). In this regard, our task was to obtain water-soluble modified chitosan, while retaining some of amino units capable of entering into the reaction of Schiff base formation, Scheme 1. The modification reaction was carried out for various periods of time (6; 12; 18; 20; 22; 24 h) in order to determine conditions when the modified chitosan becomes water-soluble (the ratio of modified and unmodified units). As can be seen from Figure 1, after 24 h the process of modifying chitosan by succinic anhydride slows down significantly, practically reaches a plateau. From the data, Table 1, we have received that water-soluble chitosan characterized by degree of substitution (DS) at least 70%, while about 60% of succinyl units are introduced, and about 25% of amino units remain unmodified. It is not possible to significantly increase the number of amino units in N-succinyl chitosan, since in this case the product will not dissolve in water.

**Figure 2:**
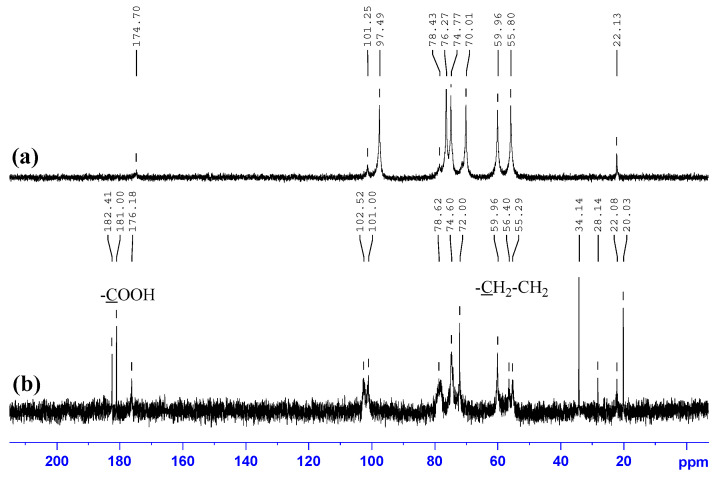
^13^C NMR spectra of: (**a**) HW-CTS; (**b**) HW-SCTS.

The ^13^C NMR spectrum of native chitosan, Figure 2a, contains signals with following chemical shifts (ppm): 22.15 (–NH(CO)CH_3_); 55.92, 60.01 (C-2, C-6); 71.93 (C-3); 74.77 (C-5); 78.43 (C-4); 97.49 (C-1); 174.70 (–NH(CO)CH_3_). The structure of modified sample, N-succinyl chitosan (SCTS), was confirmed by ^13^C NMR spectra, Figure 2b. In contrast to the ^13^C NMR spectrum of native chitosan, the spectrum of SCTS, modified for 24 h, shows the appearance of a set of bands at 34.1 and 32.7 ppm corresponding to –CH_2_-CH_2_- functionalities and a new signal of the –COONa group at 181.02 ppm, as well as shift of the carbon band of the C=O carbonyl group by 2 ppm.

Since the properties of the hydrogel are influenced by the molecular weights of polymers, LW-CTS was also modified by succinic anhydride in 24 h with producing of LW-SCTS.

The characteristics of modified chitosans: HW-SCTS—DS = 74%, M_w_ = 150 kDa; LW-SCTS—DS = 70%, M_w_ = 30 kDa.

In the work [19] N-succinyl chitosan was synthesized from chitosan with higher molecular weight (450 kDa), but authors do not describe the characteristics of the modified chitosan—the degree of modification and molecular weight.

### 2.2. HAD Synthesis and Characterization

**Scheme 2:**
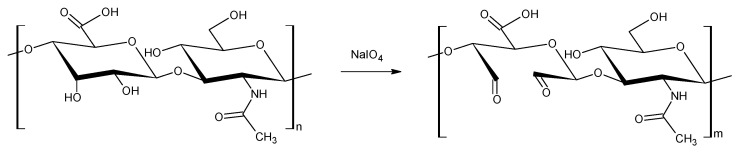
Synthesis of hyaluronic acid dialdehyde.

The oxidation of hyaluronic acid (HA) was performed using NaIO_4_ for 24 h introducing dialdehyde functionalities in several HA dimer units, Scheme 2, resulting in a simultaneous ring opening of the glucuronic acid [20].

**Figure 3:**
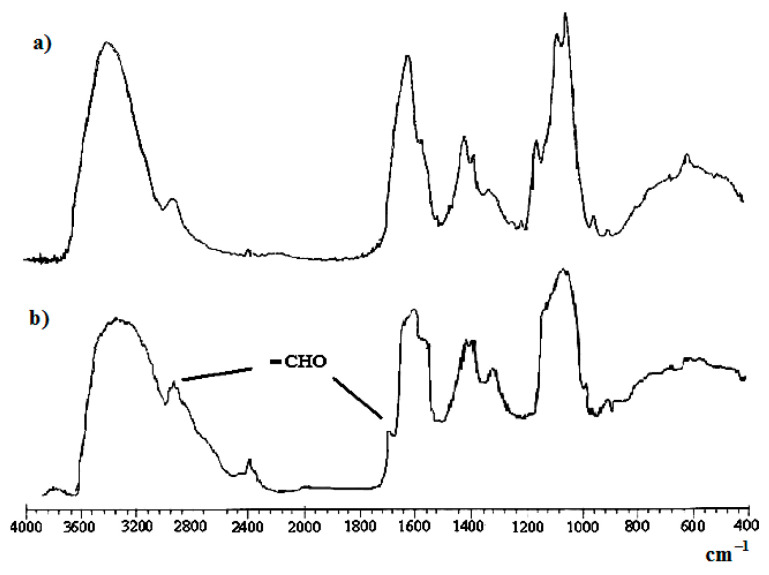
FTIR-spectra of films: (**a**) native HA; (**b**) HAD.

Aldehyde formation was proven by Fourier-transform infrared (FTIR) spectroscopy with the apparent presence of a shoulder at 1725 cm^−1^ (vibrations of C=O groups) beside the fingerprint area, Figure 3.

**Figure 4:**
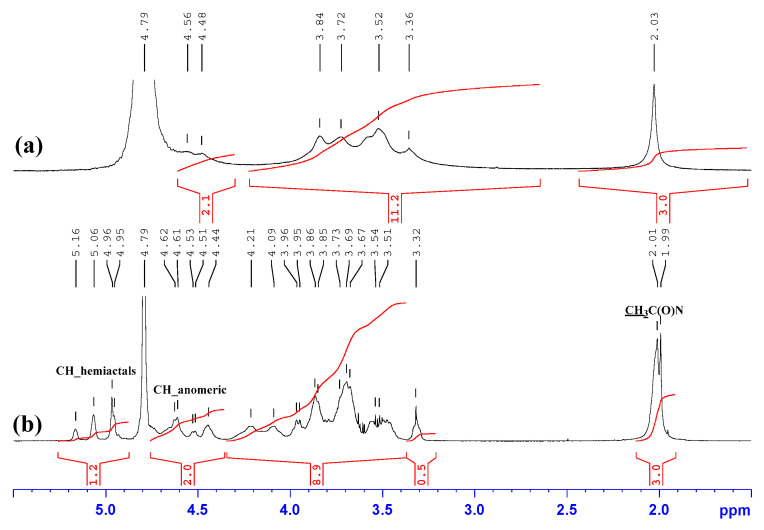
^1^H NMR spectra of: (**a**) native HA, (**b**) HAD. Solvent: D_2_O.

An indication of the successful implementation of the oxidation reaction of hyaluronic acid by sodium periodate is the appearance of three signals in the range of 4.9–5.5 ppm, Figure 4, which are associated with hydrated aldehyde groups that exist in the solution [21]. We estimated the degree of substitution of hyaluronic acid dialdehyde (HAD) by comparing the integral intensities of the signals of the hydroxyl groups of the aldehyde hydrated form with the signal of the methyl group of the acetamide fragment in the ^1^H NMR spectra and DS was about 55%.

An important fact of the oxidation reaction of hyaluronic acid by sodium periodate is a significant decrease of molecular weight of the polymer for 24 h, from 1600 kDa for native hyaluronic acid to 14 kDa for hyaluronic acid dialdehyde (HAD).

### 2.3. Characterization of SCTS—HAD Hydrogels

Hydrogels were prepared by mixing equal volumes of HAD solution (C = 1 wt.%) and SCTS solution (C = wt. 4%), prepared in phosphate-buffered saline (pH = 7.4). Such prepared hydrogels on the base of N-succinyl chitosan and hyaluronic acid dialdehyde can be categorized as in situ forming hydrogels because of the short formation time. The yield of gel-fraction is 100%, without separation of the dispersion medium.

**Figure 5:**
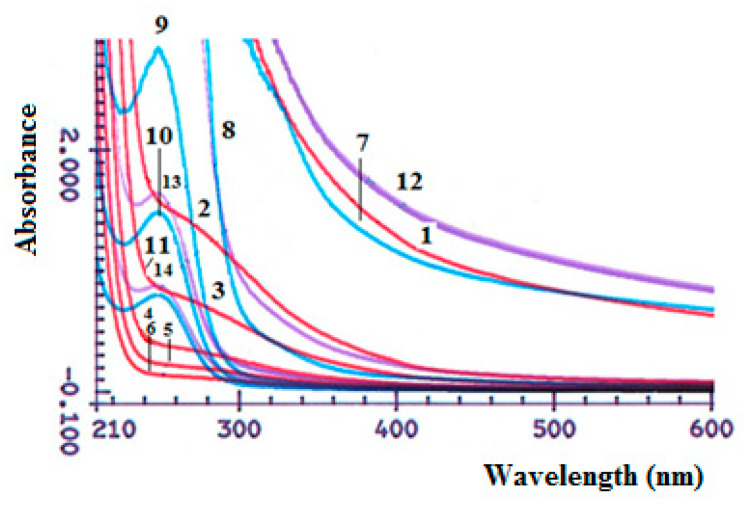
UV-Vis absorption spectra of SCTS solutions, concentration wt.%: 4.0 (1), 2.0 (2), 1.0 (3), 0.4 (4), 0.2 (5), 0.1 (6); HAD solutions, concentration wt.%: 1.00 (7), 0.50 (8), 0.10 (9), 0.05 (10), 0.03 (11); mix SCTS/HAD = 1:1: hydrogel—SCTS 4 wt.%, HAD 1 wt.% (12), solutions—SCTS 0.4 wt.%, HAD 0.1 wt.% (13), SCTS 0.2 wt.%, HAD 0.05 wt.% (14).

When mixing concentrated solutions of HAD and SCTS, an increase in the absorption intensity is noted in UV-vis absorption spectra, Figure 5, which is associated with the formation of hydrogel and changings of physicochemical properties of the polymer’s mixture, while mixing of diluted solutions is not lead to formation of hydrogel. There is no absorption maximum in the spectra of dilute SCTS solutions. While in the spectrum of HAD, the absorption maximum is determined at λ = 248 nm, characteristic of carbonyl groups (aldehyde), which is absent in the electronic spectrum of native hyaluronic acid.

**Figure 6:**
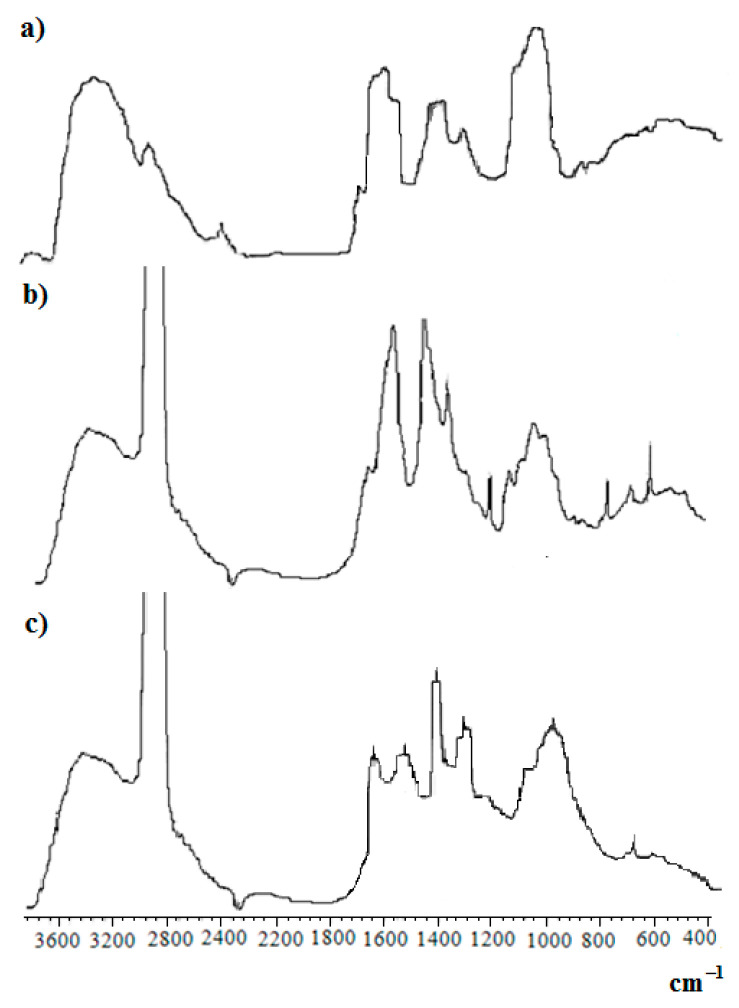
FTIR-spectra of: (**a**) film HAD; (**b**) SCTS in vaseline oil; (**c**) freeze-dried HAD—SCTS hydrogel in vaseline oil.

In the FT-IR spectra of the SCTS—HAD freeze-dried hyfrogel, Figure 6, a band appears at 1635 cm^−1^, which is apparently associated with the formation of azomethine bonds (Schiff base).

**Figure 7:**
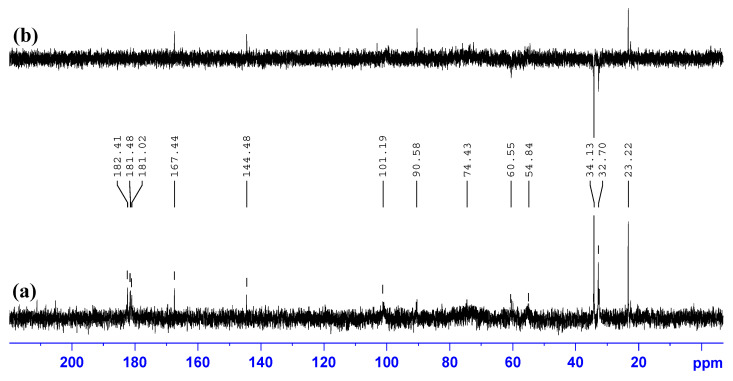
^13^C NMR spectrum (**a**) and DEPT-135 spectrum; (**b**) of SCTS—HAD hydrogel. Solvent: phosphate-buffered saline (pH = 7.4) on the base of D_2_O.

In the ^13^C NMR spectrum of the SCTS—HAD hydrogel, Figure 7, prepared in phosphate-buffered saline (pH = 7.4) on the base of D_2_O, a new signal appears in the region of 167.44 and 144.48 ppm, belonging to the two different carbons of the azomethine bonds.

**Figure 8:**
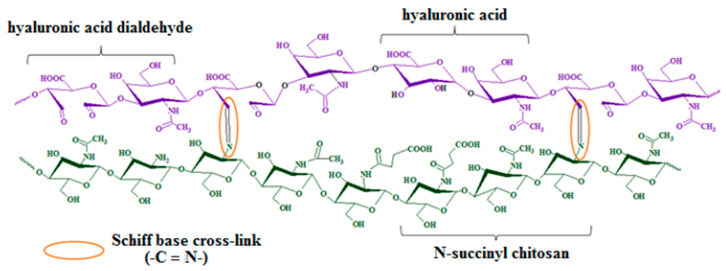
Schematic representation of hydrogel on the base of N-succinyl chitosan and hyaluronic acid dialdehyde.

Thus, the chemical cross-linking of SCTS amino units and HAD aldehyde groups leads to the formation of azomethine bonds in hydrogel in mild conditions, Figure 8. Time of gelation depends on concentration of polymers and its molar ratio. The minimum gelation time is observed with a 4-fold excess of SCTS and varies from 3 to 300 s. When using HAD excess, the hydrogel is not formed.

### 2.4. Loading of SCTS—HAD Hydrogels by Cytostatics

**Figure 9:**
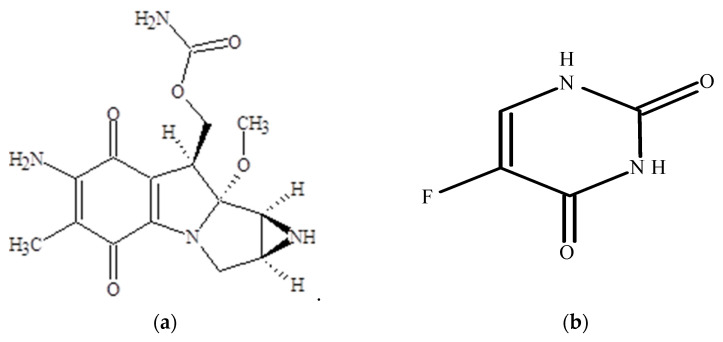
Chemical structures of cytostatics: (**a**) mitomycin C, (**b**) 5-fluorouracil.

Cytostatics, such as mitomycin C and 5-fluorouracil, are applied in oncology and ophthalmology, Figure 9. Mitomycin C (M_W_ = 334 g/mol) is a cytostatic drug from the group of antitumor antibiotics, soluble in water (0.5 mg/mL). This medicine is used directly on the one hand for the treatment of upper gastrointestinal and breast cancers. On the other hand, in eye surgery, where mitomycin C concentration of 0.02% (6 × 10^−7^ mol/mL) is applied topically to prevent scarring during glaucoma filtration surgery and to prevent opacification after PRK or LASIK. The third area of application is in esophageal and tracheal stenosis, when the application of mitomycin C to the mucous membrane immediately after dilatation reduces restenosis by reducing the formation of fibroblasts and scar tissue. It is known, that mitomycin C undergoes rapid degradation in an acidic medium at pH < 6 or heating [22,23].

5-fluorouracil (M_W_ = 137 g/mol) is anticancer drug from the group of antimetabolites, pyrimidine antagonists. It inhibits the process of cell division by blocking DNA synthesis (due to inhibition of the activity of the enzyme thymidylate synthetase) and the formation of structurally imperfect RNA (due to the introduction of fluorouracil into its structure. It also applied in ophthalmology and oncology practice [24,25].

**Figure 10:**
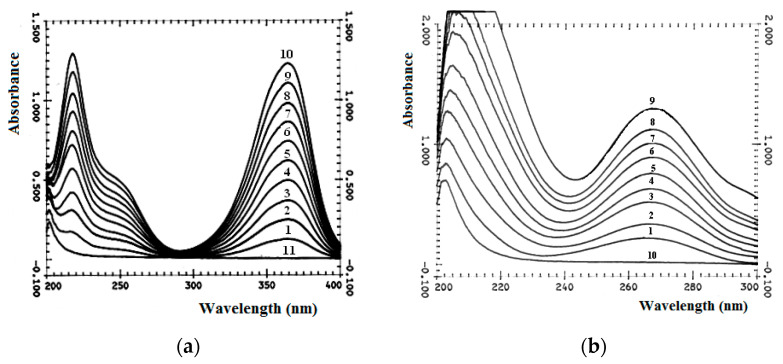
UV-Vis absorption spectra of MMC (**a**) and FU (**b**) and its mix solutions with HA. (**a**) MMC or HA 6 × 10^−5^ mol/L. MMC: HA = 1:9 (1); 2:8 (2); 3:7 (3); 4:6 (4); 5:5 (5); 6:4 (6); 7:3 (7); 8:2 (8); 9:1 (9); 10:0 (10); 0:10 (11). (**b**) FU or HA = 3 × 10^−4^ mol/L. HA: FU = 1:9 (1); 2:8 (2); 3:7 (3); 4:6 (4); 5:5 (5); 6:4 (6); 7:3 (7); 8:2 (8); 0:10 (9); 10:0 (10). Solvent: saline buffer.

**Figure 11:**
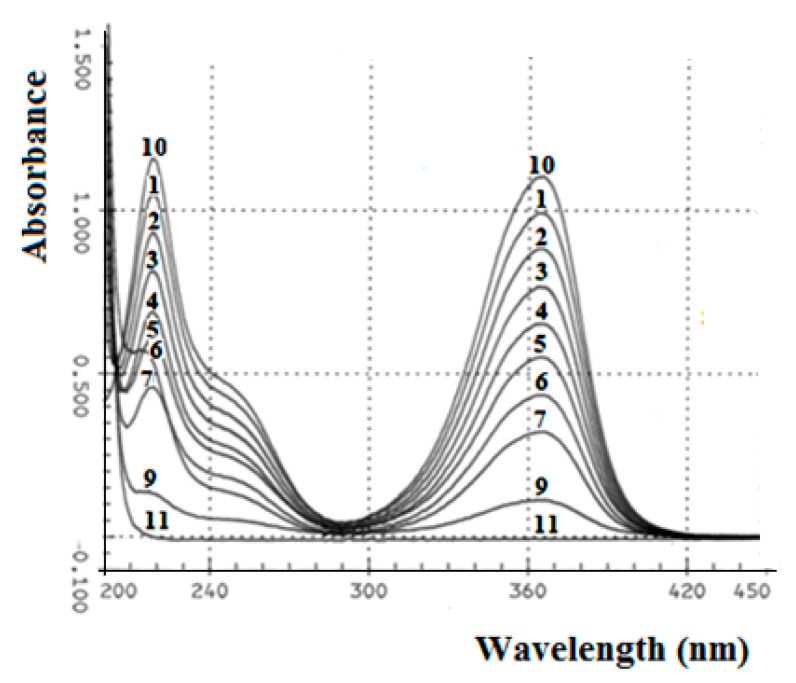
UV-Vis absorption spectra of MMC and its mix solutions with SCTS. MMC or SCTS 6 × 10^−5^ mol/L. MMC: SCTS = 1:9 (1); 2:8 (2); 3:7 (3); 4:6 (4); 5:5 (5); 6:4 (6); 7:3 (7); 8:2 (8); 9:1 (9); 10:0 (10); 0:10 (11). Solvent: saline buffer.

There are three absorption maxima in UV-Vis absorption spectrum of mitomycin C (MMC), Figure 10a, in the region of 364 nm, 250 nm and 217 nm, which correspond to two types of chromophore groups, Figure 10a and Figure 11. These are chromophores with π→π * transition of C=C bonds in the six-membered ring of MMC and with n→π * transitions of three carbonyl groups in MMC. Two carbonyl chromophores are unequally conjugated, both with electron-deficient C=C bonds of the six-membered ring of MMC, and with electron donor groups, one of which is bonded to –NH_2_, and the second to –CH_3_ and: N≡. The third carbonyl chromophore is also conjugated with the electron donor group –NH_2_ in the H_2_N–COO–CH_2_– substituent of the five-membered MMC heterocycle. The conjugation of carbonyl chromophores in MMC with π-electrons of double bonds in UV-Vis absorption spectra leads to significant bathochromic shifts of the maxima of n→π * bands to a longer wavelength part of the spectrum (a shift from 280 nm to 364 nm, which is common for carbonyl). In turn, the absorption band of the carbonyl in the composition of the substituent H_2_N–COO–CH_2_– of the five-membered heterocycle, on the contrary, undergoes a hypsochromic shift of the maximum of the n→π * transition, since it is conjugated with the isolated electron donor group –NH_2_. Absorption in the region of the 250 nm band is characteristic of benzoquinones of various degrees of substitution, types of substituents and their positions (which is observed in MMC). A chromophore with an absorption band due to the π→π * transition of electrons of the double carbon-carbon bond of the six-membered ring of MMC is observed in the electronic absorption spectrum at 217 nm.

The UV-Vis spectrum of 5-fluorouracil (FU), Figure 10b, is characterized by an absorption maximum at 270 nm, associated with the π→π * transition of carbonyl groups of the uracil ring, Figure 11b. No significant changes in hydrogel formation time were observed. So, probably, cytostatics interact with polymers in hydrogel due to weak hydrogen bonds between hydrophilic groups. In the UV-Vis absorption spectra of mixtures (Figure 10 and Figure 11), there is no appearance of new bands or significant shifts.

**Figure 12:**
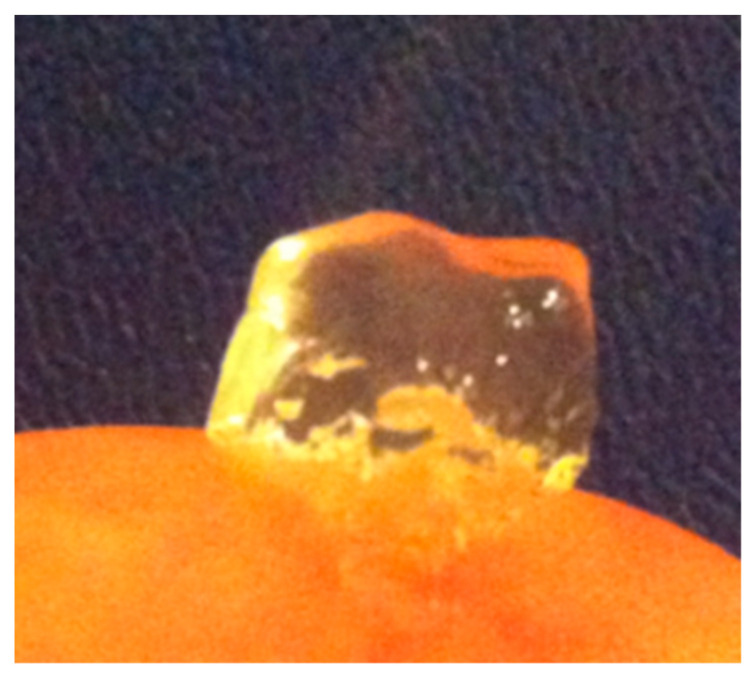
Photo of hydrogel on the base of N-succinyl chitosan and hyaluronic acid dialdehyde, containing MMC.

MMC, as well as FU, was dissolved in solution of polymer with lower molecular mass—HAD, and then resulting solution was mixed with SCTS solution. The loading of cytostatic agent (MMC or FU) into the polymer mass of the hydrogel does not visually affect its rheological properties, Figure 12, which was investigated using a rheometer.

**Figure 13:**
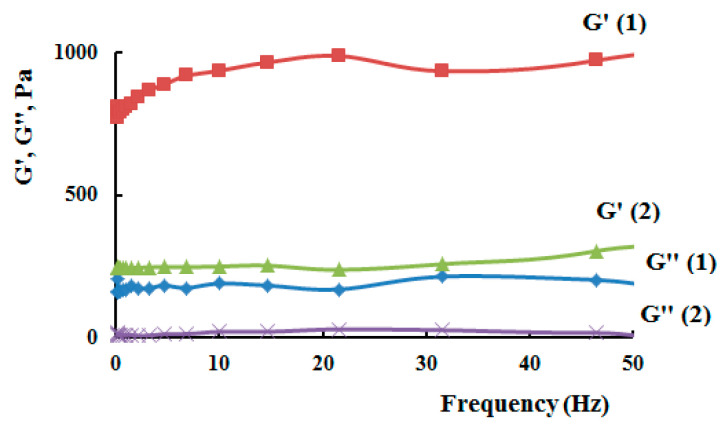
Storage modulus (G’) and loss modulus (G’’) as a function of applied frequency for SCTS–HAD hydrogels, containing MMC, where 1—HW-SCTS, 2—LW-SCTS.

The rheological data of hydrogels on the base of N-succinyl chitosan and hyaluronic acid dialdehyde, loaded with cytostatics, showed the same modulus values as hydrogels on the base of N-succinyl chitosan and hyaluronic acid dialdehyde without drugs, Figure 13, Table 2. The hydrogels exhibited typical viscoelastic behavior, as both the storage modulus and loss modulus increased with oscillating frequency. G’ was consistently greater than G” over the whole range of frequency, suggesting a general dominance of the elastic response of the gels to deformation over a broad time scale. Furthermore, both G’ and G” showed an increase with molecular weight of N-succinyl chitosan in the hydrogels, which was probably due to the improvement in the network structure of these samples and increased cross-link density.

In the study [19] hybrid hydrogels based on N-succinyl chitosan and dialdehyde starch were characterized by rheological tests (G’ from 2 kPa to 12 kPa). It can be seen that, the storage modulus values for hybrid hydrogels having high SCTS content are much higher compared to that measured samples with high dialdehyde starch contents. Hydrogels on the base of N-succinyl chitosan and dialdehyde hyaluronic acid have lower value G’, which is probably due to the lower molecular weight of the used N-succinyl chitosan, however, the resulting hydrogels retain their shape.

**Figure 14:**
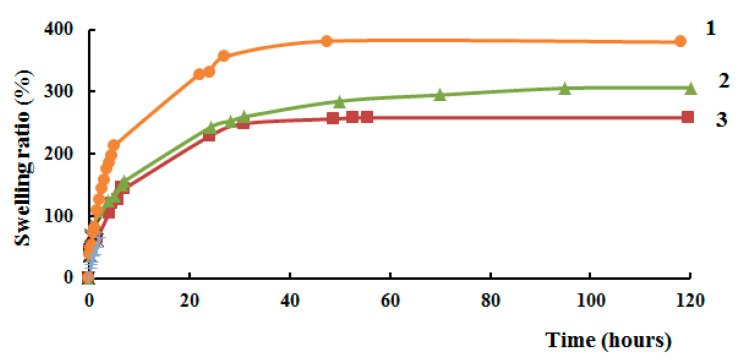
Swelling ratio of hydrogels as a function of time in normal saline, where 1—LW-SCTS-HAD, 2—HW-SCTS-HAD, 3—HW-SCTS-HAD, containing MMC.

The increase in the weight of the swollen hydrogel is directly related to the swelling period, Figure 14. After a long incubation time, the weight of the swollen hydrogel increases. The swelling coefficient of hydrogel samples rapidly increased in the first hours, which is mainly due to the large number of free adsorption sites on the pore surface of these hydrogels. Subsequently, the rate of their growth slowed down until the equilibrium water-absorbing capacity was reached. With an increase in the molecular weight of N-succinyl chitosan in the hydrogel, the swelling ratio also decreases, since longer macromolecular chains form a denser structure due to a decrease of the number of terminal units. The higher the molecular weight of N-succinyl chitosan, the more protonated amino groups are present that can be ionized. High crosslinking density limits swelling of hydrogels. This may be due to the fact that the high density of cross-linking between aldehyde and amino groups gives the hydrogel film a higher stability and hydrophobicity, increases resistance to osmotic pressure.

### 2.5. In Vitro Diffusion of Cytostatics from Polymer Matrixes

**Figure 15:**
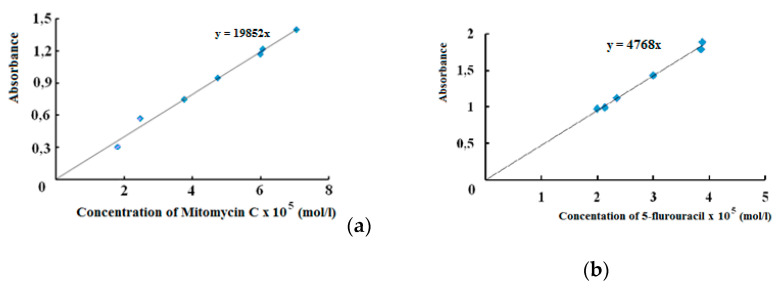
Calibration curves of cytostatics: (**a**) MMC at λ = 364 nm; (**b**) FU at λ = 268 nm.

**Figure 16:**
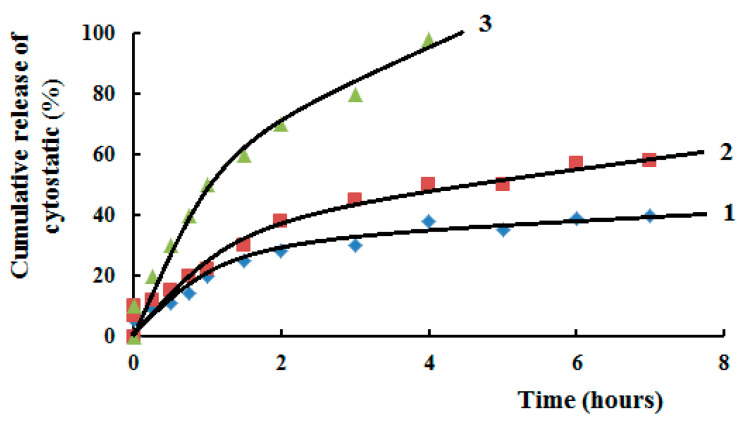
Kinetic curves of cytostatics diffusion from polymer matrix in normal saline, where 1—HW-SCTS—HAD—MMC, 2—LW-SCTS—HAD—MMC, 3—HW-SCTS—HAD—FU. Temperature 37 °C.

Using calibration curves, Figure 15, diffusion of cytostatics from polymer matrixes on the base of SCTS—HAD hydrogels was investigated. It was shown, Figure 16, that in first hours MMC released from hydrogel faster, then release rate slows down and becomes plateu. The kinetics of MMC release is influenced by the molecular weight of SCTS. Using of more high-molecular polymer in hydrogel composition slows down drug release that deals with mesh size of 3D polymer network. While FU released from hydrogel faster because of its small molecule.

The obtained hydrogels showed a prolonged release of cytostatic to the achievement of equilibrium swelling, which is probably associated with low degrees of swelling. It has been shown that hydrogels with swelling degrees >1000% release the drug at an almost constant rate, without reaching plateau region [26]. While FU released from hydrogel faster, then MMC, because of its small molecule size.

## 3. Materials and Methods

### 3.1. Materials and Reagents

High molecular weight hyaluronic acid (1600 kDa) was purchased from Sigma Aldrich, Germany; high (175 kDa) and low (35 kDa) molecular weight grades of chitosan, abbreviated as HMW-CTS and LMW-CTS were acquired from Bioprogress, Voronezh, Russian Federation. The following reagents were purchased from Merck, Germany, Darmstadt: succinic anhydride, sodium periodate, lactic acid, ethylene glycol, acetic acid, ninhydrin.

### 3.2. Synthesis of SCTS

The modification of chitosan macromolecules was carried out by succinic anhydride according to reported procedure [19] with slight modifications. 0.5 g chitosan was dissolved in 40 mL lactic acid solution (C = 5%) under magnetic stirring. 1.5 g of succinic anhydride was dissolved in 160 mL of methanol and added dropwise to the solution of polymer (with equimole ratio). The reaction mixture was stirred for predetermined time intervals (6; 12; 18; 20; 24 h). The temperature of reaction was 20 °C. The modified chitosan was isolated using precipitation process by adjusting the pH of mixture solution to 8. The resultant precipitate SCTS was filtered off, washed off in ethanol, then acetone, re-dissolved in distilled water, dialyzed for 3 days, and dried in vacuum.

### 3.3. Characterization of SCTS

^1^H and ^13^C NMR spectroscopy was used to prove the formed N-succinyl chitosan. The degree of substitution (DS) of modified chitosan (SCTS) was determined by ninhydrine analysis [27].

### 3.4. Synthesis of HAD

The oxidation of hyaluronic acid (HA) was carried out according to Ref. [20] with some modifications. In short, 0.5 g of HA was dissolved in 50 mL of distilled water. 2.5 mL of solution of 0.5 M sodium periodate was added dropwise to an aqueous solution of HA and reaction mixture was kept for 24 h at 20 °C. To inactivate unreacted sodium periodate, 0.5 mL of ethylene glycol was added, and the mixture was stirred for another 2 h. For purification, the reaction mixture was dialyzed against distilled water for 3 days and freeze-dried.

### 3.5. Characterization of HAD

FTIR-spectrometry (Tensor-27 BRUKER, Bremen, Germany) was used to prove the formed aldehyde functions in macromolecules of HAD. To determine the quantity of aldehyde groups, ^1^H NMR spectra were used. By comparing the integrals of protons of the methyl group of acetamide fragment with the total of the signals of protons of the aldehyde hydrates for HAD [28].

### 3.6. Gelation of SCTS with HAD

The investigated polymers (HAD, SCTS, HA, CTS) were dissolved in distilled water, phosphate-buffered saline (pH = 7.4) or normal saline (pH = 7) at various polymer concentrations. The different volumes of resulting polymer solutions were mixed at room temperature. Solution of cytostatic (mitomycin C or 5-fluorouracil) was added to solution of lower molecular weight polymer (HAD) and then mixed with SCTS solution. The formation of the hydrogel was monitored by the loss of fluidity of the system over time by visual examination.

### 3.7. Rheology

The rheological properties of the hydrogels SCTS-HAD, containing cytostatics, were investigated using a stress-controlled modular dynamic rheometer Haake Mars III (Karlsruhe, Germany) at 25 °C.

The viscoelastic properties of hydrogels can be characterized by the following parameters: dynamic viscosity η*, storage (elastic) modulus G′, loss (viscous) modulus G′′ and complex shear modulus G*. The storage modulus accounts for the elastically stored energy, while the loss modulus accounts for the irreversibly energy dissipating through viscous flow [21].

The network parameters can be calculated using the frequency independent plateau modulus *G’_p_*. The rubber elastic theory (RET) makes it possible to calculate the number of cross-links *ν_e_* and the distance between two entanglement points (average mesh size) *ξ* in accordance with the equations [21,29]:

Number of cross-links:

$$\nu_{e}=\frac{G_{p}^{’}N_{A}}{RT}$$

Average mesh size:

$$\xi =\frac{1}{\sqrt[{3}]{\nu_{e}}}$$

### 3.8. Swelling Ratio Study

Swelling ratios of prepared hydrogels were observed in normal saline. Specific amount of each hydrogel was immersed in buffer solution. At specific time interval, hydrogels were taken out, blotted with filter paper and weighted. The same procedure was repeated three times for all hydrogels and the average value was adapted to measure the swelling ratio (*SR*, %) as stated:

$$SR=\frac{W_{0}-W_{t}}{W_{0}}\times 100\%$$

where, *W_0_* is the initial weight of hydrogel, *W_t_* is the weight of the hydrogel in the moment *t*.

### 3.9. In Vitro Release Study

A sample of hydrogel, containing cytostatic, was immersed in normal saline at temperature 37 °C. The kinetics of changes in absorbance of solution was recorded by UV-spectroscopy (Shimadzu UV-3100, Tokyo, Japan). The concentration of cytostatics was calculated using calibration curves. The standard curves of mitomycin C and 5-fluorouracil were generated by analyzing standard solutions of known concentrations.

## 4. Conclusions

Hydrogel matrices composed of N-succinyl chitosan of different molecular weights and hyaluronic acid dialdehyde as drug delivery systems for cytostatics, 5-fluorouracil or mitomycin C, were prepared and investigated for their potential applicability for surgical treatment of glaucoma or cancer. By using of ^13^C NMR spectroscopy it was established the formation of azomethine bonds (Schiff base) due to interactions of amino groups of N-succinyl chitosan and aldehyde groups of hyaluronic acid dialdehyde. Chemically cross-linked structure of obtained hydrogel provides elastic properties of matrix. Obtained formulations were evaluated for their ability to form hydrogels, rheological properties and drug release in vitro. The molecular weight of N-succinyl chitosan was found to modulate drug release profiles of the formulations. N-succinyl chitosan with the highest molecular weight (150 kDa) combined hyaluronic acid dialdehyde showered more prolonged release of mitomycin C compared to the hydrogel with N-succinyl chitosan with lower molecular weight or hydrogel containing 5-fluorouracil, which is probably due to the size of the drug molecules. In summary, chemically cross-linked hydrogels on the base of N-succinyl chitosan and hyaluronic acid dialdehyde provided promising future for various biomedical applications in drug delivery.

## 5. Patent

Bikbov, M. M.; Khusnitdinov, I. I.; Sigaeva, N. N.; Kolesov, S. V.; Vildanova, R. R. The drainage for glaucoma treatment. RU 2610407 C, 2017.

## Figures and Tables

**Table 1:** Characteristic of chitosan before and after modification by succinic anhydride depending on the reaction time.

Reaction Time, h	Content of Units, %		Molar Ratio R-NH_2_: R-COOH	Degree of Substitution of SCTS, %	Solubility of SCTS in Water
	R-NH_2_	R-COOH			
	84				Insoluble
6	47	37	0.8: 1.0	44	Insoluble
12	34	50	1.0: 1.5	60	Insoluble
18	27	57	1.0: 2.1	67	Insoluble
20	25	59	1.0: 2.3	70	Soluble
22	24	60	1.0: 2.5	72	Soluble
24	23	61	1.0: 2.6	74	Soluble

**Table 2:** Characteristic of SCTS—HAD hydrogels, containing MMC.

SCTS, wt.%	M_w_ (SCTS), kDa	Dynamic Viscosity η*, Pa∙s	Plateau Modulus G’p, Pa	Distance between Two Entanglement Points ξ, nm
4	150	380	240	26
4	30	1250	780	17
